# 

**Published:** 1897-12-18

**Authors:** 


**Notices of Births, Deaths, and Marriages** are inserted in this column at a charge of 2s. 6d. for an announcement not exceeding four lines.

### NOTICE TO CORRESPONDENTS.

All MS., Letters, Books for Review, and other matters intended for the Editor should be addressed The Editor, "The Hospital," The Hospital Building, 28 & 29, Southampton Street, Strand, W.C.

The Editor cannot undertake to return rejected MS., even when accompanied by stamped directed envelope.

**Notice to Advertisers and Subscribers.**

All Advertisements, Orders for Copies of The Hospital, and Business Communications should be addressed to **THE MANAGER, (Not to The Editor),** The Hospital, The Hospital Building, 28 & 29, Southampton Street, Strand, London, W.C.

The Cost of Subscription, post free, is as follows:—Per week, 2½d.; per quarter, 2s. 8d.; per half-year, 5s. 3d.; per annum, 10s. 6d. Foreign:—Per annum, 15s. 2d.; payable, in all cases, in advance.

### BOOKS RECEIVED.

Poor Law Officers' Journal.

"Treatment of Imbeciles and Epileptics." By Dr. J. M. Rhodes and Alderman A. McDougall.

Walter Scott.

The Contemporary Science Series. "Sleep: Its Physiology, Pathology, Hygiene, and Psychology." By Marie de Manacéïne.
"The Psychology of the Emotions." By Th. Ribot.

T. Fisher Unwin.

"Sir James Young Simpson." By H. Laing Gordon. Masters of Medicine Series.

**Periodicals and Pamphlets Received.**—Proceedings of the Sixth International Conference of the Red-Cross Societies, held at Vienna, September, 1897; Nursing Notes, Guy's Hospital Gazette, Medical Press, African Critic, Literary Digest, Australian Sun, Woman's Signal, Electrical Review, Knowledge, Humanitarian, Leeds Hospital Magazine, The Quarterly Magazine of James Murray's Royal Asylum, Perth.

### THE STUDENT OF MEDICINE.

### APPOINTMENTS.

Dr. W. C. Bosanquet, appointed Assistant Physician to the Victoria Hospital, Chelsea. J. J. G. Brown, M.D., F.R.C.P.E., F.R.S.E., appointed an Assistant Physician to the Edinburgh Royal Infirmary. J. W. Carr, M.D.Lond., M.B., B.S., appointed Physician to the Victoria Hospital, Chelsea. H. Chatterton, M.R.C.S., L.R.C.P., appointed Assistant Medical Superintendent to the St. Pancras Union Infirmary, Highgate. J. E. H. Davies, M.R.C.S., L.R.C.P., appointed Medical Officer for the No. 6. District of the Wrexham Union. S. R. Deane, L.R.C.S.Irel., L A.H.Dub., appointed Medical Officer to the Children's Homes of the Lincoln Union. G. Dickson, F.R.C.P.E., L.R.C.S.E., L.F.P.S.G., appointed Medical Officer of Health to the town of Hay, and Medical Officer for the Radnorshire District of the Hay Union. C. J. Glasson, M.R.C.S.Eng., L.R.C.P.Lond., appointed Medical Officer of Health to the Ellesmere Urban and Rural District Councils. W. V. Griffith, L.R.C.S., L.R.C.P., appointed Medical Officer for the No. 2 District of the Wrexham Union. J. C. Harcourt, M.R.C.S., L.R.C.P., appointed Assistant Medical Officer to the Infirmary of the Lambeth Union. F. J. L. Hart, M.B., C.M.Edin., appointed Divisional Surgeon to the Metropolitan Police. G. W. C. Hodges, M.R.C.S.Eng., L.R.C.P.Lond., appointed Medical Officer for the Second District of the Bridgnorth Union. Dr. J. H. Jones, appointed Medical Officer for the First District of the Launceston Union. T. R. Jones, M.D.Aberd., M.B., M.R.C.S., appointed Consulting Physician to the Victoria Hospital, Chelsea. J. F. Johns, M.D.Durh., D.P.H., R.C.P.S.Eng., appointed Medical Officer of Health to the Cheltenham Rural District Council. J. L Johnstone, M.R.C.S., L.R.C.P., appointed Medical Officer of Health to the Upholland Urban District Council. R. Lake, F.R.C.S., appointed Assistant Surgeon to the Royal Ear Hospital, Soho. A. C. Latham, B.A.Oxon, M.B., B.Ch., appointed Assistant Physician to the Victoria Hospital, Chelsea. G. M. McKie, M.R.C.S., L.R.C.P., appointed Junior House Surgeon to the Seamen's Branch Hospital, Albert Dock, E. J. M'Kie, M.B., C.M., appointed Medical Officer for the Springburn District of the Barony Parish. R. L. Meade-King, M.D.Durh., appointed an Honorary Physician to the Taunton and Somerset Hospital. A. Mirza, M.B., B.Sc.Edin., F.R.I.P.H., appointed Medical Officer of Health to Chaderghat Muncipality, in addition to the Medical Officership to the City of Hyderabad Municipality. D. E. Moss, appointed Medical Officer of the Workhouse of the Wrexham Union. H. M. Murray, M.D., F.R.C.P.Lond., appointed Physician to the Victoria Hospital, Chelsea. A. A. Mussen, M.D.Dubl., appointed House Physician to the Hospital for Women, Soho Square, W. O. Osborne, L.R.C.P.Lond., M.R.C.S.Eng., appointed Medical Officer of Health to the Bexhill Urban District Council. C. J. Palmer, L.S.A.Lond., appointed Medical Officer for No. 2 District of the Mansfield Union. J. R. Prior, M.B.Durh., M.R.C.S., appointed Surgeon to the Seamen's Hospital Society's Dispensary, East India Dock Road, E. Dr. Richards, appointed Medical Officer and Public Vaccinator of the Swinhope District of the Caistor Union. C. H. Tattersall, L.R.C.P.Lond, M.R.C.S.Eng., appointed Medical Officer of Health for Salford. W. G. Westcott, M.R.C.S.Eng., L.R.C.P.Lond., appointed House Surgeon to the West Sussex, East Hants, and Chichester Infirmary. P. M. Yearsley, F.R.C.S., appointed Assistant Surgeon to the Royal Ear Hospital, Soho.

### PASS LIST.

### University of London

M.B. EXAMINATION: OCTOBER, 1897.

Examination for Honours.

Medicine.—First Class: M. Dixon, B.Sc. (gold medal), University College; C. H. Fagge (scholarship and gold medal), Guy's Hospital. Third Class: C. W. Alford, Middlesex Hospital; B. Dyball, St. Thomas's Hospital; C. Dykes, University College; F. W. Robertson, St. Bartholomew's Hospital; A. W. Sikes, B.Sc., St. Thomas's Hospital; F. H. Thiele, B.Sc., University College; E. J. Toye, B.Sc., St. Bartholomew's Hospital; W. P. Walker, Owens College and Manchester Royal Infirmary; R. J. Warrington, Owens College and Manchester Royal Infirmary.

